# Stereotactic Radiotherapy Ablation and Atrial Fibrillation: Technical Issues and Clinical Expectations Derived From a Systematic Review

**DOI:** 10.3389/fcvm.2022.849201

**Published:** 2022-05-03

**Authors:** Jessica Franzetti, Stefania Volpe, Valentina Catto, Edoardo Conte, Consiglia Piccolo, Matteo Pepa, Gaia Piperno, Anna Maria Camarda, Federica Cattani, Daniele Andreini, Claudio Tondo, Barbara Alicja Jereczek-Fossa, Corrado Carbucicchio

**Affiliations:** ^1^Department of Radiation Oncology, European Institute of Oncology (IEO) IRCCS, Milan, Italy; ^2^Department of Oncology and Hemato-Oncology, University of Milan, Milan, Italy; ^3^Department of Clinical Electrophysiology and Cardiac Pacing, Centro Cardiologico Monzino IRCCS, Milan, Italy; ^4^Department of Electronics, Information and Biomedical Engineering, Politecnico di Milano, Milan, Italy; ^5^Cardiovascular Computed Tomography and Radiology Unit, Centro Cardiologico Monzino IRCCS, Milan, Italy; ^6^Unit of Medical Physics, European Institute of Oncology (IEO) IRCCS, Milan, Italy; ^7^Department of Biomedical and Clinical Sciences “Luigi Sacco”, University of Milan, Milan, Italy; ^8^Department of Biomedical, Surgical and Dental Sciences, University of Milan, Milan, Italy

**Keywords:** systematic review, stereotactic arrhythmia radio ablation (STAR), atrial fibrillation, arrhythmias, stereotactic body radiotherapy (SBRT), particle beam radiotherapy, target motion

## Abstract

**Aim:**

The purpose of this study is to collect available evidence on the feasibility and efficacy of stereotactic arrhythmia radio ablation (STAR), including both photon radiotherapy (XRT) and particle beam therapy (PBT), in the treatment of atrial fibrillation (AF), and to provide cardiologists and radiation oncologists with a practical overview on this topic.

**Methods:**

Three hundred and thirty-five articles were identified up to November 2021 according to preferred reporting items for systematic reviews and meta-analyses criteria; preclinical and clinical studies were included without data restrictions or language limitations. Selected works were analyzed for comparing target selection, treatment plan details, and the accelerator employed, addressing workup modalities, acute and long-term side-effects, and efficacy, defined either by the presence of scar or by the absence of AF recurrence.

**Results:**

Twenty-one works published between 2010 and 2021 were included. Seventeen studies concerned XRT, three PBT, and one involved both. Nine studies (1 *in silico* and 8 *in vivo*; doses ranging from 15 to 40 Gy) comprised a total of 59 animals, 12 (8 *in silico*, 4 *in vivo*; doses ranging from 16 to 50 Gy) focused on humans, with 9 patients undergoing STAR: average follow-up duration was 5 and 6 months, respectively. Data analysis supported efficacy of the treatment in the preclinical setting, whereas in the context of clinical studies the main favorable finding consisted in the detection of electrical scar in 4/4 patients undergoing specific evaluation; the minimum dose for efficacy was 25 Gy in both humans and animals. No acute complication was recorded; severe side-effects related to the long-term were observed only for very high STAR doses in 2 animals. Significant variability was evidenced among studies in the definition of target volume and doses, and in the management of respiratory and cardiac target motion.

**Conclusion:**

STAR is an innovative non-invasive procedure already applied for experimental treatment of ventricular arrhythmias. Particular attention must be paid to safety, rather than efficacy of STAR, given the benign nature of AF. Uncertainties persist, mainly regarding the definition of the treatment plan and the role of the target motion. In this setting, more information about the toxicity profile of this new approach is compulsory before applying STAR to AF in clinical practice.

## Introduction

Atrial fibrillation (AF) is one of the most common cardiac arrhythmias, with an estimated number 8.8 million of affected subjects in Europe. As the prevalence of AF increases with age, it is expected to affect approximately 18 million in the European Union by 2060 (1) and more than 8 million people in the United States by 2050 (2). In addition, the incidence of AF increases in patients with cancer having an incidence of 3.7 per 1,000 person year, also due to medical oncology treatments (3).

Despite being benign in nature, AF represents a well-recognized independent risk factor for stroke (4) and has been associated with potentially severe medical conditions including heart disease (5) and chronic kidney disease (6). Moreover, a substantial proportion of eligible patients are undertreated with medical therapy (7) and 74.6% of the patients (5) are symptomatic despite ongoing medical therapy. Drugs can also have significant side effects such as an increased risk of bleeding; all these features determine a worsened quality of life in patients with AF (8). Based on the presentation, duration, and spontaneous termination of AF episodes, five types of AF can be distinguished: first diagnosed, paroxysmal (self-terminating, in most cases within 48 h), persistent, long-standing persistent (continuous AF lasting for ≥1 year), and permanent AF (AF that is accepted by the patient and physician) (9). As AF frequently originates from an electric trigger located in the pulmonary veins, this site is the main therapeutic target of an ablation procedure defined as “pulmonary veins isolation” (PVI) (10). Based on further empirical evidence, the left-posterior atrial wall has been added to this target (11). A wide variety of approaches for PVI, including point-by-point radiofrequency ablation or cryoballoon ablation (9), has been described. Recently, pulsed-field ablation has been introduced as an innovative technique for the ablation of AF. It is based on the induction of cell death by the electric field (electroporation), has shown good preliminary results in terms of safety and efficacy (12, 13). Overall, the efficacy of these procedures reaches 70% in patients with paroxysmal AF and 50% in those with persistent AF (14), while the percentage of severe related complications approximates 3.5% (15). In addition, a significant proportion of patients require more than one procedure to achieve the permanent restoration of sinus rhythm (SR) (16).

While alternative techniques are available, including ethanol, needle, and bipolar ablation, they are not without disadvantages or side effects, including the uncertainty of properly and completely damaging the target (17), cardiac perforation and tamponade (18), or the inability to appropriately hit deep and large substrates (19).

Other than the well-known applications in cancer, radiation therapy has been used for the treatment of benign medical conditions, showing both satisfactory efficacy and a good safety profile (20–22).

In the last 5 years, multiple studies have investigated the potential of stereotactic arrhythmia radio ablation (STAR): most of the literature is about the treatment of recurrent ventricular tachycardia (VT) and involves both conventional linear accelerator (CLA) (23, 24) and radiosurgery Cyberknife^®^ (CK, Accuray, Sunnyvale, CA, United States) accelerator (25–27). The safety and efficacy of this new therapeutic opportunity seem to be good in both cases. Moreover, some preclinical studies (28, 29) have used particle beam therapy (PBT) for cardiac ablation: being able to selectively spare the most critical structures is a clear advantage and might arguably open up to the future possibility of re-irradiations.

An increasing body of literature has focused on intracardiac malignancies undergoing stereotactic radiosurgery, and on its possible related side effects (30–32). Similarly, dosimetric studies on heart irradiation have been published in the last years (33, 34). A significant issue of cardiac radiosurgery may be the long-term effects of radiation on myocardial, conduction, valvular, and other cardiac tissues. These concerns can be at least partially addressed by the study of long-term toxicity in lymphoma (35) and centrally located lung treatment (36).

Although photons are the most known form of energy in radiation therapy, PBT (both heavy ions and protons) are an emerging alternative to conventional treatments. Advantages of this form of radiotherapy are the favorable physical characteristics and the major relative biological effectiveness (RBE), especially when referring to carbon ion radiotherapy (37). As a consequence, studies favoring the role of stereotactic PBT have been developed (38) over the last couple of years.

Given the lack of comparable works, this article aims to review the current evidence on the feasibility and efficacy of external beam radiotherapy for AF and to provide radiation oncologists and cardiologists with a practical overview of this theme.

## Materials and Methods

In compliance with the preferred reporting items for systematic reviews and meta-analyses (PRISMA) (39, 40), literature research was performed in November 2021.

**FIGURE 1:**
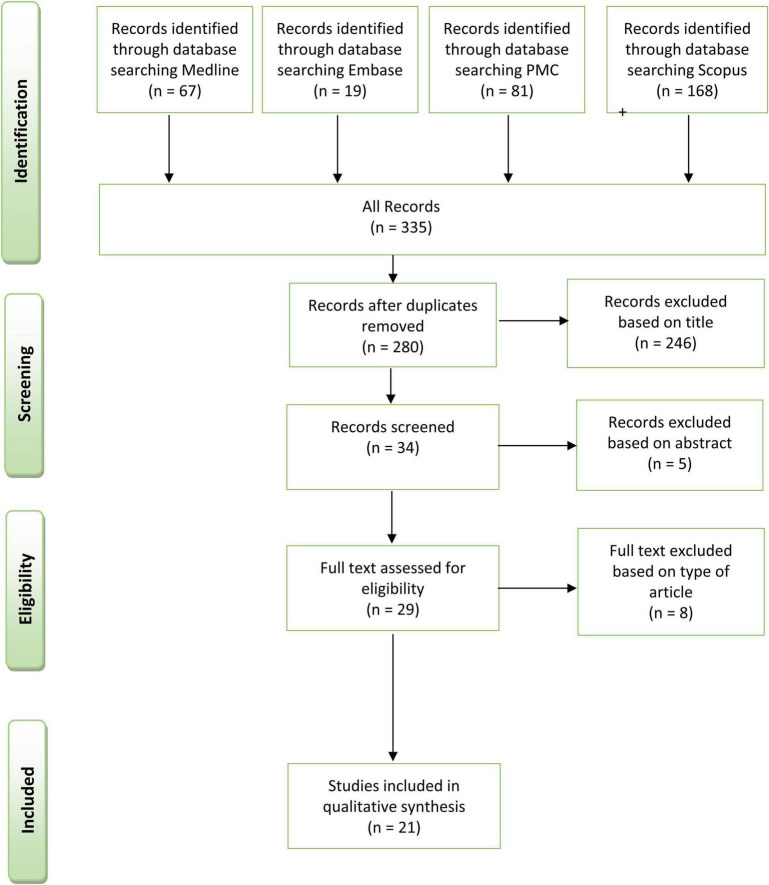
Preferred reporting items for systematic reviews and meta-analyses flow-chart.

Articles were researched in multiple database sources: NCBI PubMed, EMBASE, PMID, and Scopus. The strings of research employed and the PRISMA’s checklist are available in Supplementary Material 1. The PRISMA flow diagram for article selection is illustrated in Figure 1.

Both preclinical and clinical studies were considered; no data restrictions or language limitations were applied. The inclusion of gray literature was allowed. Studies whose focus were other forms of arrhythmias (i.e., ventricular and nodal) were considered out of the scope of this work and were therefore excluded.

An independent re-assessment was performed by a second reviewer; in case of any disagreement, a third reviewer was engaged. Selected works were independently screened by two reviewers; whenever disagreement occurred regarding the inclusion criteria, a third reviewer was called to resolve the discrepancy.

Summary and definition of the radiation oncology-related terms are available in Supplementary Material 2.

## Results

Following reviewing and duplicate removal, a total of 21 articles presented from 2010 to 2021 was included in the analysis.

They consisted of one and 8 preclinical studies on treatment plans for animals and humans treatments, respectively, 8 preclinical studies on animal models, and 4 clinical studies on human subjects. Here follows an overview of selected articles, categorized according to the above-mentioned criteria.

### Preclinical Studies

#### Animals Subjects

The first study on the *in vivo* cardio ablation for AF was performed in 2010 by Sharma et al. (41). Overall, preclinical studies were conducted on healthy animals subjects, with 26 mini-pigs; in only 3 studies also canines were considered (42–44), for a total number of 27 cases (Table 1). Average or median doses could not be calculated for preclinical works due to a lack of information in some of the included studies.

**TABLE 1:** Main characteristics of the preclinical studies on animals included in the analysis.

Study	Energy	N° subjects	Subjects	Total dose (Gy)	N° of fractions	Target	Fiducials	Accelerator	Respiratory motion control	Cardiac motion control	Delivered plan	Follow-up (months)	Efficacy	Toxicity
Blanck et al. (45)	XRT	9	Mini-pigs	15–35	1	RSPV	N/A	CLA	Yes	Yes	Yes	6	Dose ≥32.5 Gy	No
Bode et al. (46)	XRT	8	Mini-pigs	23–40	1	RSPV	No	CLA	Yes	Yes	Yes	6	Dose ≥30 Gy	Dose ≥37.5 Gy
Chang et al. (44)	XRT	7	Canines	33	1	WACA	N/A	CLA	Partially	No	Yes	2–4	50%	Pericardial effusion
Gardner et al. (42)	XRT	4	Canines, mini-pigs	20–35	1	PVA	Yes	CK	Yes	Yes	Yes	5	N/A	No
Maguire et al. (47)	XRT	2	Mini-pigs	25–35	1	PVA	Yes	CK	Yes	Yes	Yes	6	Yes	Trace MVR
Sharma et al. (41)	XRT	4	Mini-pigs	38–40	1	LPV	Yes	CK	Yes	Yes	Yes	1–6	Yes	No
Zei et al. (43)	XRT	19	Canines, mini-pigs	15–35	1	RSPV	Yes	CK	Yes	Yes	Yes	3–6	Dose ≥25 Gy	Min. reduction EF

*CLA, conventional linear accelerator; CK, Cyberknife; EF, ejection fraction; LPV, left pulmonary vein; MVR, mitral valve regurgitation; N/A, not available; PVA, pulmonary vein antra; RSPV, right superior pulmonary vein; WACA, wide area circumferential ablation; XRT, photon radiotherapy.*

In most cases, animals underwent general anesthesia and received ablation in a single fraction delivered by CK. For treatments delivered by CK, fiducials (both gold seeds and catheter tips) were necessary to evaluate target motion. A CLA was used in 3 cases (44–46). In almost all the articles, both cardiac and respiratory motions were considered, except for Chang et al. (44) who acquired four-dimensional computed tomography (4DCT) only in case of the large respiratory amplitude of the animal, a single phase scan was considered and performed in others. The same authors tried to use masks in 2 dogs and had to change immobilization systems to a vacuum cushion during simulation CT.

The target of the procedure was different across the studies: some works evaluated either left pulmonary veins alone (41) or the right pulmonary veins (43, 45, 46), while 3 studies considered both targets (42, 47). Zei et al. considered only the right superior pulmonary vein as a target because of the excessive respiratory motion of the left superior pulmonary vein in the canine model (43). The follow-up ranged between 1 and 6 months. Efficacy of radiotherapy ablation was generally confirmed at doses up to about 25–30 Gy; side effects (i.e., bronchial-mediastinal fistula with pneumonia and sepsis) were observed in one mini-swine 1 month after irradiation when the delivered dose exceeded 37.5 Gy (46). Moreover, one animal experienced a myocardial infection following fiducial marker placement (43) and another pericardial effusion (44). Mild side effects were mitral valve regurgitation after the procedure in one case (42), one mild reduction of ejection fraction (43), and electrocardiographic, self-limiting abnormalities on T wave after anesthesia (4 animals) (43). On the other hand, one animal died due to pericarditis after electrophysiological study (45). Findings in animal studies were usually evaluated by means of electroanatomic mapping, MRI (46), or anatomopathological study after sacrificing the subjects.

A different approach was chosen by Gardner et al. (42), where the implantable metal oxide semiconductor field-effect transistor (MOSFET) dose verification system and the thermoluminescent dosimetry in pulmonary vein antra (PVA) isolation through CK technology were compared. The authors observed that the implantation method adopted for the MOSFET system shows a better concordance than thermoluminescent dosimetry since it appears not to be affected by body fluids. However, the difference between the measured and the predicted doses in the MOSFET system still accounts for almost 10% when the acceptance threshold has been set at 5% by previous studies (48, 49). The authors hypothesized that a degree of uncertainty might derive from the impossibility to track the dose verification system during treatment delivery.

#### Dosimetric Studies

In the category of dosimetric studies, a total of 122 treatment plans on both photon radiotherapy (XRT) and PBT were considered, with a median dose of 25 Gy (Table 2).

**TABLE 2:** Main characteristics of the dosimetric photon and particle beam-based studies included in the analysis.

Study	Energy	N° subjects	Subjects	Total dose (Gy)	N° of fractions	Target	Fiducials	Accelerator	Respiratory motion control	Cardiac motion control	Delivered plan	Follow-up (months)	Efficacy	Toxicity
Blanck et al. (50)	XRT	46	Humans	25	1	PVA	Yes	CK	Yes	Yes	No	N/A	N/A	N/A
Constantinescu et al. (58)	PBT	14	Humans	25–40	1	WACA	No	AA	Yes	Yes	No	N/A	N/A	N/A
Gardner et al. (51)	XRT	4	Humans	16–25	1	PVA ± LPW	No	CK	N/A	N/A	No	N/A	N/A	N/A
Ipsen et al. (55)	XRT	6	Humans	30	1	PVA	No	MRIL	Yes	Yes	No	N/A	N/A	N/A
Lehmann et al. (60)	PBT	3	Pigs	30–40	1	RSPV-LAJ	Yes	AA	Yes	Yes	Yes	6	Yes	No
Lydiard et al. (53)	XRT	15	Humans	50	5	PVA	No	CLA	Partially	Partially	Dynamic phantom	N/A	N/A	N/A
Ren et al. (61)	XRT/PBT	11	Humans	25	1	WACA	No	AA/CLA	Yes	Yes	No	N/A	N/A	N/A
Richter et al. (59)	PBT	3	Pigs	30–40	1	RSPV-LAJ	Yes	AA	Yes	Yes	No	N/A	N/A	N/A
Xia et al. (52)	XRT	20	Humans	50	5	PVA	No	CLA	No	No	No	N/A	N/A	N/A

*AA, adron accelerator; CLA, conventional linear accelerator; CK, Cyberknife; EF, ejection fraction; LPV, left pulmonary veins; LPW, left posterior wall; MRI, magnetic resonance imaging; MRIL, MRI-Linac, MRI-linear accelerator; MVR, mitral valve regurgitation; N/A, not available; PBT, particle beam therapy; PVA, pulmonary vein antra; RSPV, right superior pulmonary vein; RSPV-LAJ, right superior pulmonary vein-left atrial junction; WACA, wide area circumferential ablation; XRT, photon radiotherapy.*

A dosimetric study is a preclinical work in which subjects undergo simulation CT without experiencing radiation treatment or in which the treatment plan is delivered to a phantom. These permit the evaluation of dosimetry in the target region and organs at risk, avoiding any toxicity.

Meanly treatment plans consisted of one single fraction and were delivered with CK in 2 cases (50, 51). Conversely, in the other studies, a greater dose was planned with a CLA: 50 Gy in 5 fractions (10 Gy/fraction), according to the biological effective dose (BED) (52, 53). According to Xia et al. (52), a radiobiological modeling study (54) was used for BED calculation with an alpha/beta ratio of 3 Gy; in the second study, Lydiard et al. (53) did not explain how BED was evaluated and which alpha/beta ratio considered. The prescription dose was delivered to a dynamic phantom only in the study by Lydiard et al. (53). Specifically, the authors registered the respiratory profiles of 3 healthy patients and subsequently associated the recorded profiles to the phantom to deliver plans differing in complexity. The first was created using a dynamic conformal arc and a 3 mm target volume (TV) margin expansion, one using volumetric modulated arc therapy (VMAT) plan, a restricted number of monitor unit and a 3-mm TV margin expansion, the other VMAT plans with TV margin expansions of 0, 3, and 5 mm, respectively. All dynamic plans were compared with the static ones, and the superiority of multileaf collimator (MLC) tracking over tracking without MLC was demonstrated, with a minor failure percentage appreciated through a gamma failure rate, and a better TV dose coverage.

Only Ipsen et al. (55) involved MRI-linear accelerator (MRI-Linac) in their work: they evaluated the role of real-time MRI target localization and efforted the treatment planning for cardiac radiosurgery with MRI-Linac on 6 male volunteers.

The above-described preclinical studies considered PVA as the only target of irradiation. Only Gardner et al. (51) compared 2 different target extensions: PVA and PVA plus left posterior wall (LPW); the last one was optimized to spare mitral valve annulus, right coronary, and circumflex arteries. Better compliance with radiation therapy oncology group limits was observed in the second target set, with the purpose to reduce the dose to the ventricles where most cardiac adverse events after radiation therapy would originate (56).

Overall, fiducials were implanted only by Blanck et al. (50); in this work, the authors compared a spectrum of different tracking systems: the partially invasive one, such as a catheter in the right atrial septum (temporary fiducials), CK marker-less tracking system for lung tumors (XSight^®^ Lung, Accuray) or ultrasound tracking (50). In the same article, Blanck et al. (50) described a prevalidation, contouring study comprising a series of 133 patients’ CT scans: esophagus segmentation revealed that in 50% of the cases the organ is directly in contact with the target, similarly to transcatheter ablation (57).

#### Particle Beam Therapy

Four studies focused on PBT, and carbon ions were used in all cases: 2 works were dosimetric (58, 59) and one reported on *in vivo* dosimetry on animals (60). The last one (61) compared intensity-modulated proton therapy with XRT delivered through VMAT and helical tomotherapy.

Constantinescu et al. (58) evaluated 9 and 5 CT scans of complete respiratory and cardiac cycles, respectively: they planned 25–40 Gy single fraction carbon ion treatments involving intensity-modulated particle therapy (IMPT). Authors defined the importance of respiratory and heartbeat motions with a lesion displacement of, respectively, ≤2 cm and <6 mm; in this last case a worsening in dose coverage (V95 < 90%) was registered. Carbon ion beam rescanning was used to improve dose coverage.

The same rescanning technique was employed also by Lehmann et al. (60) to reduce the interference between the scanning motion of the beam and the target motion, the so-called “interplay.” In their work, carbon ion irradiation in different TV was evaluated: a 30–40 Gy single fraction treatment was delivered on the right superior pulmonary vein-left atrial junction (RSPV-LAJ) of 3 pigs; one of the 3 animals was irradiated with a lower dose of 30 Gy to spare esophagus (due to specifical anatomy of the animal), the others with 40 Gy. All the treatments were delivered with in-beam positron emission tomography (PET) to verify the correct deposition of carbon ions during irradiation. In the end, they evaluated apoptotic markers employing the Western blot technique with anti-caspase-3, antitubuline, and horseradish peroxidase-conjugated secondary antibodies. As a result, they found that an increase of these markers occurred 3 months after the irradiation, but 6 months after the treatment all the markers turned negative. With the same dataset, Richter et al. (59) evaluated 17 treatment plans (3 on RSPV-LA, 14 on other targets) with ECG-based-4D-dose reconstruction, showing higher safety with respect to cardiac structures and efficient dose verification.

The most recent article included on PBT, Ren et al. (61), evaluated dosimetric properties of intensity-modulated proton therapy in comparison with VMAT and tomotherapy treatment planning; the prescription dose was 25 Gy in all plans. The proton-based technique resulted in a significantly reduce dose in surrounding tissues, compared to photon-based ones, in patients with AF.

### Clinical Studies

Three of the selected studies considered human subjects, with a total number of 6 patients (Table 3). The first clinical work is a case report by Monroy et al. (62) on a 59-year-old man with symptomatic AF suffering from adverse effects caused by antiarrhythmic drugs and an ischemic stroke in oral anticoagulant therapy. The need of performing catheter manipulation within the left atrium, which is required by classical PVI, was judged as a contraindication to a catheter-based procedure. Therefore, a radio ablation was proposed by the cardiologist, and the patient underwent radiosurgery, delivered by CK in a single fraction, with a prescription dose to pulmonary veins of 25 Gy to the 71% isodose line. Details on the use of fiducials were not reported, and the details of cardiac motion control. Respiratory motion was compensated by synchrony image guidance during the whole course of treatment delivery. Six months after the procedure the patient developed a permanent AF requiring him to restart the medical therapy. An MRI was performed 1 year the after procedure and a late enhancement was recorded at the radio-ablated structure, which may correspond to the development of a scar.

**TABLE 3:** Main characteristics of the clinical studies included in the analysis.

Study	Energy	N° subjects	Total dose (Gy)	N° of fractions	Target	Fiducials	Accelerator	Respiratory motion control	Cardiac motion control	Delivered plan	Follow-up (months)	Efficacy	Toxicity
Monroy et al. (62)	XRT	1	25	1	PVA	N/A	CK	Yes	N/A	Yes	12	No	No
Qian et al. (63)	XRT	2	25–35	1	WACA	Yes	CK	Yes	Yes	Yes	48	50%	No
Shoji et al. (64)	XRT	3	22–30	1	WACA	Yes	CK	Yes	Yes	Yes	24	No	No

*CK, Cyberknife; LPV, left pulmonary veins; N/A, not available; PVA, pulmonary vein antra; WACA, wide area circumferential ablation; XRT, photon radiotherapy.*

A second study [Qian et al. (63)] involved 2 patients with symptomatic AF who had refused a catheter procedure and had agreed to an experimental non-invasive ablation. Both had undergone a fiducial placement and a subsequent simulation contrast-enhanced CT scan. A prescription dose of 25 Gy was delivered through a CK accelerator in both cases. Patients were followed for 24 months (patient 1) and 48 months (patient 2), showing the absence of any significant treatment-related side effects. Six months after irradiation, patient 1 developed persistent AF, leading to permanent medical therapy. Conversely, the second patient had no AF recurrences during the entire follow-up. Only the second patient performed a pre- and post-ablation MRI, showing evidence of a scar at the radiosurgery site after 1 year.

The most recent article in the clinical area has been published by Shoji et al. (64): 3 oncologic patients with refractory AF were treated with a target dose of 25–30 Gy in a single fraction delivered by CK. The TV was represented by a “box” lesion set including a circumferential wide-area ablation (WACA) set around pulmonary veins and the maximum follow-up was 24 months. One patient died 4 days after the procedure due to oncologic disease. The autopsy revealed evidence of fibroblasts and fibrogenesis in the region of radio-ablated tissues. On the other two patients, who remained in AF, clear evidence of clinical efficacy cannot be found: authors encountered some limitations as a consequence of the second patient’s refusal to undergo electrograms of LPW recorded from the esophagus. However, the third patient underwent this exam and no atrial potentials were seen from the esophageal electrogram recordings after radio ablation. This evidence suggests an electrical block, which is the clinical goal of the procedure. No acute or late effects were registered during follow-up.

### Gray Literature

Two of all the articles selected were gray literature: the first was the preclinical study of Rahimian et al. (65) which included 3 patients’ treatment plans for a 25 Gy single fraction therapy. The most recent study, Gregucci et al. (66) is currently enrolling patients, and results are not yet available. All the studies considered PVA as TV. No information about efficacy or toxicity is now available from all this literature but it suggests the increasing interest in this particular topic.

## Discussion

### Main Evidence

Results from our work show the application of STAR for AF. A prescription dose of at least 25 Gy in a single fraction is necessary to have good efficacy despite an acceptable toxicity profile.

The major cause of failure of traditional catheter ablation of AF is incomplete circumferential vein isolation (9). It is worth considering that, according to the existing literature on catheter ablation, the choice of the target (11) and the circumferential scar (67) is essential to obtain an effective procedure. Target selection appears to have the same importance in non-invasive cardio-ablation procedures, as confirmed by target heterogeneity among considered studies (see section “Results”).

Target motion control, involving fiducials or other simulation strategies (4DCT and cardio-CT or electroanatomic mapping) is deemed necessary to improve the accuracy of the procedure.

It is worth saying that, despite the interest in the topic, a limited number of humans has currently undergone STAR for AF and only 2 articles including more than one patient have been published (63, 64). In the study of Quian et al. (63), efficacy was observed in one of 2 treated patients, but no detail on treatment plan features was provided by the authors; moreover, 2 different pathways of preprocedural and follow-up exams were applied, which cannot be considered as being completely comparable. The absence of toxicity was the only shared feature between the patients included. In the study of Shoji et al. (64), no acute or late effects were observed; nevertheless, the choice to select oncologic patients makes it more difficult to evaluate the endpoint of efficacy. Even if clinical efficacy on human subjects is difficult to be defined in a limited sample, fibrosis (63, 64), or electrical block (64) was observed in the radio-ablated area in both studies. A similar finding, obtained by an MRI exam, was recorded in the case report (62). All the above-mentioned evidence may be interpreted as the confirmation of the radio-ablation lesion.

In conclusion, available evidence reports acceptable tolerability of the cardio-ablation treatment on humans; further analyses, together with the newest results coming from the current “gray literature,” however, are deemed necessary to reach the highest level of efficacy.

### Validation of Stereotactic Arrhythmia Radio Ablation With Regard to Different Experimental Settings

We observed a prevalence of preclinical studies, the majority of which involved mini pigs. This choice can be explained by their relative growth stability and the consequent capability of weight maintenance during follow-up. Three of the analyzed studies also considered a canine model (42–44). However, regardless of the chosen animal models (68), transferability concerns for clinical applications in humans exist. Significant examples may be the incomplete pericardium of dogs (47) or the different cardiac chambers anatomy and number of pulmonary veins in humans and canines (44). Specifically, these anatomical peculiarities could affect respiratory and cardiac target motions, which are essential parameters in treatment planning.

When evaluating the preclinical studies on animals it has been shown that the efficacy is higher when mini pigs (41, 47) are treated as compared with canines (44) or mixed samples (42).

Total prescription doses in the considered works ranged from 15 to 50 Gy/fraction and the minimum dose threshold for efficacy was 25 Gy. Most of the studies encompassed stereotactic radiosurgery delivered in a single fraction except for few articles describing 5-fraction treatments with a dose of 10 Gy/fraction (total dose: 50 Gy). This comparability is based on the BED which was calculated by the authors (52) using a radiobiological model to avoid the overestimation of the total dose resulting from the linear-quadratic BED calculation when the dose is greater than 8–10 Gy (69). Of note, even if BEDs were considered comparable, the heart tissue is a late responder and its alpha/beta ratio is about 3 Gy (31, 52) with the consequence that the effect may be superior with higher doses delivered in a single fraction than with lower fractionated doses.

A discrete number of studies based on the treatment plan evaluation or delivery of the treatment on a dynamic phantom can be found in the literature: even if they appear to be more acceptable from the ethical standpoint, someone may question if the evidence acquired from these studies are comparable, in terms of efficacy and safety, with those acquired from the clinical setting; in some cases (53), authors started from a study of cardiac and respiratory montions on healthy patients, raising the question whether the respiratory and cardiac motions are really comparable in healthy patients with AF, as discussed below.

### Role of the Target Motion

The role of the target motion was furthermore discussed in almost all studies. This topic gains importance since the natural motion of the organs influences not only the myocardial or the conduction tissue around the target but also the other organs at risk, the most important one appearing to be the esophagus. The problem of organ motion was solved by some authors (50, 59, 60) by adopting different fiducials such as seeds or catheters, whereas other ones did not (46, 51–53). The presence of fiducials makes the tracking useful in the positioning of the patient and in the reduction of the margin of error due to cardiac and respiratory motion, but it implies the use of tools that are against the peculiar nature of the procedure in terms of non-invasiveness.

Grimm et al. (70) and Abelson et al. (71) faced the problem of organs at risk doses by reviewing literature and patients’ data, respectively, and elaborated dosimetric tables as references for the colleagues’ work.

In the end, it is important to remember how it is possible that a dilated heart with AF appears to have less movement than a healthy one (61, 72): so, it is also possible that all dosimetric studies on healthy subjects are not completely suitable for the patients with real-AF and more investigations could be necessary.

At last, it is worth noticing the interesting application of MRI to approach the problem of target motion (55, 73): on the one hand, by quantifying target motion ranges on MRI, on the other hand by analyzing the dosimetric benefits of margin reduction assuming the application of real-time motion compensation.

Supporting this hypothesis, a recent article by Lydiard et al. (74), not included in the selection, investigated the feasibility of non-invasive MRI-guided tracking of cardiac-induced target motion in AF cardiac radio ablation by comparing a direct tracking method and 2 indirect tracking methods (tracking indirect left atrial or other targets). They suggested the applicability of non-invasive MRI-guided tracking, showing a potential improvement in treatment efficacy.

### Particle Beam Therapy: Pros and Prospectives

**FIGURE 2:**
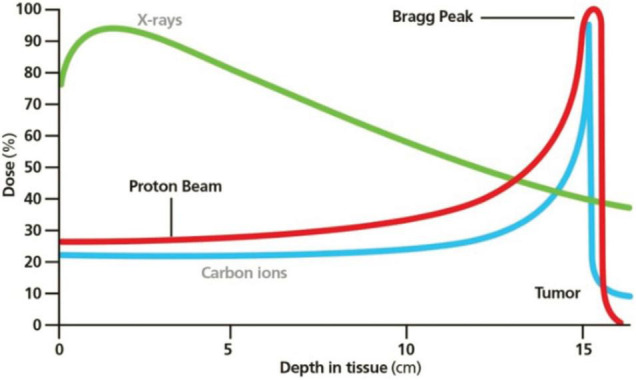
Bragg peak in PBT. Reprinted from Mustapha et al. (81) with the permission of AIP Publishing.

Both XRT and PBT involve ionizing radiations, but the second one can deliver its maximum dose at a specific depth (Bragg peak, Figure 2) to the TV while no dose in the surrounding tissues (75).

Carbon ion should be particularly indicated for the aim of cardio ablation because of the favorable RBE (three times as much as the photons’ one) and the possibility of smaller beam foci and less lateral scattering.

In the included articles, pencil beams were used to better modulate the beam on the TV; the limit of these thin rays is a major sensibility to motion and setup errors, then the correct position of the beam’s distal edge remains unknown (75). Ren et al. (61) decided to study this phenomenon in their work using a cardiac motion scan from a patient case. Nevertheless, beam rescanning and 4D dose calculation (58) or the use of in-beam PET can reduce the problem (60).

An interesting biological hypothesis about the effectiveness of carbon ions in arrhythmia ablation was formulated by Amino et al. (76): they studied the role of the upregulation of connexin-43, a protein expressed during myocardial remodeling in myocardial infarction or cardiac hypertrophy. This remodeling effect on gap junctions may reduce the conduction of the arrhythmia through myocardial tissue.

Although photons and carbon ions are so different, according to the articles selected, the time to detect a scar in an anatomopathological analysis is similar and it spans from weeks to months. As further evidence, the process of fibrosis and scar creation starts after the activation of the apoptotic cascade (77, 78), according to Lehmann et al.’s results (60).

### Use of Stereotactic Arrhythmia Radio Ablation in Atrial Fibrillation Versus Ventricular Tachycardia

During the evaluation of the efficacy and safety of STAR in AF, some considerations about the comparison between AF and VT are necessary. First, it is worth underlying that specific peculiarities characterize the anatomical and structural substrate for AF as for VT, which reflect in different treatment approaches and need to safeguard surrounding healthy structures. For these reasons, some assumptions that have been preliminarily validated in the field of VT may not be true for AF. Ventricular arrhythmias, that may deserve STAR, are usually life-threatening; patients present with recurrent and/or refractory VTs and are not eligible for conventional approaches or these have proven ineffective. In this clinical setting, STAR represents a promising option, thus more risks, even unknown ones, are allowed. To the best of our knowledge, in literature few severe adverse events, definitely correlated to STAR, are reported. In particular, one patient died of esophagopericardial fistula after 9 months from STAR: of note, the patient had previous bypass surgery with a gastroepiploic artery that might have contributed to this severe adverse event (79); few clinically relevant or symptomatic radiation-induced pericarditis and pericardial effusion and a gastropericardial fistula 2 years after STAR were recorded (80).

Being AF a benign arrhythmia, more attention to the safety rather than the efficacy of STAR is mandatory.

In this setting, more information about the toxicity profile of this new approach is compulsory before applying STAR to AF in clinical practice; this is also the reason why not many clinical articles are available in the literature so far.

### Strengths and Limitations

All the above-mentioned works and other already published reviews discuss every type of tachydysrhythmias without a specific focus on AF. The strength of this review is the specificity of the topic treated: stereotactic radio ablation of AF through both XRT and PBT. In this regard, we would like to underline once again that these considerations do not necessarily apply to other patients’ conditions (e.g., non-oncological patients).

The main limitations of this work are the relative paucity of works, which is in line with the novelty of the field, and the low evidence of available literature. Moreover, given the nature of our work (qualitative rather than quantitative synthesis), and the relative paucity of studies, it was not possible to fully estimate publication bias—if any—through a funnel plot. To at least account for such potential weakness, gray literature was also included. As a matter of fact, while these works are not peer-reviewed, good-quality gray literature is a source of up-to-date information on ongoing clinical efforts.

## Conclusion

Stereotactic radio ablation is an innovative non-invasive procedure already in use for ventricular cardiac arrhythmias. Radio ablation of AF, with a prescription dose at least of 25 Gy, might be considered among the future therapeutic option for AF, especially when an interventional ablation procedure is contraindicated or proved ineffective.

Carbon ions are a highly promising radiation technique due to their TV coverage and, at the same time, their greater capability to spare organs at risk; this may be a strong point to achieve an effective safer alternative application for the heart.

Essential issues, such as:

– duration of AF before treatment,
– target definition and motion, and
– doses delivered to the target and organs at risk,

deserve further evaluation to define proper indications and modalities to benefit the most from the use of STAR in patients with AF.

## Data Availability Statement

The original contributions presented in the study are included in the article/Supplementary Material, further inquiries can be directed to the corresponding author.

## Author Contributions

JF contributed to conception of the study and wrote the first draft of the manuscript. SV wrote the first draft of the manuscript and was the third reviewer of the literature. VC performed the literature research and contributed to write the first draft of the manuscript. CP was the first reviewer of the literature. EC, GP, and FC contributed to write sections of the manuscript. MP designed and prepared the tables. AMC was the second reviewer of the literature. DA and CT critically revised the final version. BAJ-F contributed to conception of the study and critically revised the final version. CC designed the study and contributed to wrote the first draft of the manuscript and the final revision. All authors contributed to manuscript revision, read, and approved the submitted version.

## Conflict of Interest

The authors declare that the research was conducted in the absence of any commercial or financial relationships that could be construed as a potential conflict of interest.

## Publisher’s Note

All claims expressed in this article are solely those of the authors and do not necessarily represent those of their affiliated organizations, or those of the publisher, the editors and the reviewers. Any product that may be evaluated in this article, or claim that may be made by its manufacturer, is not guaranteed or endorsed by the publisher.

## Abbreviations

4DCT: four-dimensional computed tomography
AF: atrial fibrillation
BED: biological effective dose
CK: Cyberknife
CLA: conventional linear accelerator
IMPT: intensity-modulated proton therapy
LPW: left posterior wall
MOSFET: metal oxide semiconductor field effect transistor
MRI: magnetic resonance imaging
MRI-Linac: MRI-linear accelerator
PBT: particle beam therapy
PET: positron emission tomography
PRISMA: preferred reporting items for systematic reviews and meta-analyses
PVA: pulmonary vein antra
PVI: pulmonary vein isolation
RBE: relative biological effectiveness
RSPV-LAJ: right superior pulmonary vein-left atrial junction
SR: sinus rhythm
STAR: stereotactic arrhythmia radio ablation
TV: target volume
VMAT: volumetric modulated arc therapy
VT: ventricular tachycardia
WACA: wide-area circumferential ablation
XRT: photon radiotherapy.

## References

[1] Krijthe BP Kunst A Benjamin EJ Lip GYH Franco OH Hofman A Projections on the number of individuals with atrial fibrillation in the European Union, from 2000 to 2060. *Eur Heart J.* (2013) 34:2746–51. 10.1093/eurheartj/eht280 23900699 PMC3858024

[2] Naccarelli GV Varker H Lin J Schulman KL. Increasing prevalence of atrial fibrillation and flutter in the United States. *Am J Cardiol.* (2009) 104:1534–9. 10.1016/j.amjcard.2009.07.022 19932788

[3] Jakobsen CB Lamberts M Carlson N Lock-Hansen M Torp-Pedersen C Gislason GH Incidence of atrial fibrillation in different major cancer subtypes: a nationwide population-based 12 year follow up study. *BMC Cancer.* (2019) 19:1105. 10.1186/s12885-019-6314-9 31726997 PMC6854796

[4] Wolf PA Abbott RD Kannel WB. Original contributions atrial fibrillation as an independent risk factor for stroke: the Framingham study. Stroke. (1991) 22:983–8. 10.1161/01.str.22.8.983 1866765

[5] Zoni-Berisso M Filippi A Landolina M Brignoli O D’Ambrosio G Maglia G Frequency, patient characteristics, treatment strategies, and resource usage of atrial fibrillation (from the Italian survey of atrial fibrillation management [ISAF] study). *Am J Cardiol.* (2013) 111:705–11. 10.1016/j.amjcard.2012.11.026 23273528

[6] Hart RG Eikelboom JW Brimble KS McMurtry MS. Stroke prevention in atrial fibrillation patients with chronic kidney disease. *Can J Cardiol.* (2013) 29(7 Suppl.):S71–8. 10.1016/j.cjca.2013.04.005 23790601

[7] Marzec LN Wang J Shah ND Chan PS Ting HH Gosch KL Influence of direct oral anticoagulants on rates of oral anticoagulation for atrial fibrillation. *J Am Coll Cardiol.* (2017) 69:2475–84. 10.1016/j.jacc.2017.03.540 28521884

[8] Go AS Hylek EM Phillips KA Chang Y Henault LE Selby JV Prevalence of diagnosed atrial fibrillation in adults national implications for rhythm management and stroke prevention: the anticoagulation and risk factors in atrial fibrillation (ATRIA) Study. *JAMA.* (2001) 285:2370–5. 10.1001/jama.285.18.2370 11343485

[9] Kirchhof P Benussi S Kotecha D Ahlsson A Atar D Casadei B 2016 ESC Guidelines for the management of atrial fibrillation developed in collaboration with EACTS. *Europace.* (2016) 18:1609–78. 10.1093/europace/euw295 27567465

[10] Haïssaguerre M Jaïs P Shah DC Takahashi A Hocini M Quiniou G Spontaneous initiation of atrial fibrillation by ectopic beats originating in the pulmonary veins. *N Engl J Med.* (1998) 339:659–66. 10.1056/nejm199809033391003 9725923

[11] Abozguia K Cutler MJ Ziv O. The presence of left atrial posterior wall fibrillation despite restoration of sinus rhythm after posterior box ablation. *Hear Case Rep.* (2015) 1:416–8. 10.1016/j.hrcr.2015.03.005 28491597 PMC5419696

[12] Verma A Boersma L Haines DE Natale A Marchlinski FE Sanders P First-in-human experience and acute procedural outcomes using a novel pulsed field ablation system: the PULSED AF pilot trial. *Circ Arrhythm Electrophysiol.* (2022) 15:e010168. 10.1161/CIRCEP.121.010168 34964367 PMC8772438

[13] Reddy VY Koruth J Jais P Petru J Timko F Skalsky I Ablation of atrial fibrillation with pulsed electric fields: an ultra-rapid, tissue-selective modality for cardiac ablation. *JACC Clin Electrophysiol.* (2018) 4:987–95. 10.1016/j.jacep.2018.04.005 30139499

[14] Calkins H Reynolds MR Spector P Sondhi M Xu Y Martin A Treatment of atrial fibrillation with antiarrhythmic drugs or radiofre- quency ablation: two systematic literature reviews and meta-analyses. *Circ Arrhythm Electrophysiol.* (2009) 2:349–61. 10.1161/circep.108.824789 19808490

[15] Gupta A Perera T Ganesan A Sullivan T Lau DH Roberts-Thomson KC Complications of catheter ablation of atrial fibrillation: a systematic review. *Circ Arrhythm Electrophysiol.* (2013) 6:1082–8. 24243785 10.1161/CIRCEP.113.000768

[16] Takigawa M Takahashi A Kuwahara T Okubo K Takahashi Y Watari Y Long-term follow-up after catheter ablation of paroxysmal atrial fibrillation the incidence of recurrence and progression of atrial fibrillation. *Circ Arrhythm Electrophysiol.* (2014) 7:267–73. 10.1161/CIRCEP.113.000471 24610740

[17] Sacher F Sobieszczyk P Tedrow U Eisenhauer AC Field ME Selwyn A Transcoronary ethanol ventricular tachycardia ablation in the modern electrophysiology era. *Heart Rhythm.* (2008) 5:62–8. 10.1016/j.hrthm.2007.09.012 18180021

[18] Sapp JL Beeckler C Pike R Parkash R Gray CJ Zeppenfeld K Initial human feasibility of infusion needle catheter ablation for refractory ventricular tachycardia. *Circulation.* (2013) 128:2289–95. 10.1161/CIRCULATIONAHA.113.003423 24036605

[19] Nguyen DT Tzou WS Brunnquell M Zipse M Schuller JL Zheng L Clinical and biophysical evaluation of variable bipolar configurations during radiofrequency ablation for treatment of ventricular arrhythmias. *Heart Rhythm.* (2016) 13:2161–71. 10.1016/j.hrthm.2016.07.011 27424078

[20] Pou P Biau J Verrelle P Lemaire JJ El Ouadih Y Chassin V Long-term outcomes after linac radiosurgery for benign meningiomas. *Clin Oncol.* (2020) 32:452–8. 10.1016/J.CLON.2020.02.006 32201158

[21] Zecchin M Severgnini M Fiorentino A Malavasi VL Menegotti L Alongi F Management of patients with cardiac implantable electronic devices (CIED) undergoing radiotherapy. *Int J Cardiol.* (2018) 255:175–83. 10.1016/j.ijcard.2017.12.061 29310933

[22] Riva G Alessandro O Spoto R Ferrari A Garibaldi C Cattani F Radiotherapy in patients with cardiac implantable electronic devices: clinical and dosimetric aspects. *Med Oncol.* (2018) 35:73. 10.1007/s12032-018-1126-3 29667046

[23] Cuculich PS Schill MR Kashani R Mutic S Lang A Cooper D Noninvasive cardiac radiation for ablation of ventricular tachycardia. *N Engl J Med.* (2017) 377:2325–36. 10.1056/NEJMoa1613773 29236642 PMC5764179

[24] Carbucicchio C Andreini D Piperno G Catto V Conte E Cattani F Stereotactic radioablation for the treatment of ventricular tachycardia: preliminary data and insights from the STRA-MI-VT phase Ib/II study. *J Interv Card Electrophysiol.* (2021) 62:427–39. 10.1007/s10840-021-01060-5 34609691 PMC8490832

[25] Loo BW Soltys SG Wang L Lo A Fahimian BP Iagaru A Stereotactic ablative radiotherapy for the treatment of refractory cardiac ventricular arrhythmia. *Circ Arrhythm Electrophysiol.* (2015) 8:748–50. 10.1161/CIRCEP.115.002765 26082532

[26] Robinson CG Samson PP Moore KMS Hugo GD Knutson N Mutic S Phase I/II trial of electrophysiology-guided noninvasive cardiac radioablation for ventricular tachycardia. *Circulation.* (2019) 139:313–21. 10.1161/CIRCULATIONAHA.118.038261 30586734 PMC6331281

[27] Gianni C Rivera D Burkhardt JD Pollard B Gardner E Maguire P Stereotactic arrhythmia radioablation for refractory scar-related ventricular tachycardia. *Heart Rhythm.* (2020) 17:1241–8. 10.1016/j.hrthm.2020.02.036 32151737

[28] Lehmann HI Richter D Prokesch H Graeff C Prall M Simoniello P Atrioventricular node ablation in langendorff-perfused porcine hearts using carbon ion particle therapy. *Circ Arrhythm Electrophysiol.* (2015) 8:429–38. 10.1161/CIRCEP.114.002436 25609687

[29] Amino M Yoshioka K Fujibayashi D Hashida T Furusawa Y Zareba W Year-long upregulation of connexin43 in rabbit hearts by heavy ion irradiation animal handling followed the guide for the care and use of laboratory animals. *Am J Physiol Heart Circ Physiol.* (2010) 298:1014–21. 10.1152/ajpheart.00160.2009.-A 20061548

[30] Martin AGR Coltart DJ Plowman PN. CyberKnife radiosurgery for an intracardiac metastasis. *BMJ Case Rep.* (2011):bcr0720103197. 10.1136/bcr.07.2010.3197 22707622 PMC3062820

[31] Soltys SG Kalani MY Cheshier SH Szabo KA Lo A Chang SD. Stereotactic radiosurgery for a cardiac sarcoma: a case report. *Technol Cancer Res Treat.* (2008) 7:363–8. 10.1177/153303460800700502 18783285

[32] Bonomo P Cipressi S Desideri I Masi L Doro R Iermano C Stereotactic body radiotherapy with CyberKnife for cardiac malignancies. *Tumori.* (2015) 101:294–7. 10.5301/tj.5000280 25908042

[33] Adams MJ Lipshultz SE Schwartz C Fajardo LF Coen V Constine LS. Radiation-associated cardiovascular disease: manifestations and management. *Semin Radiat Oncol.* (2003) 13:346–56. 10.1016/S1053-4296(03)00026-2 12903022

[34] Gagliardi G Constine LS Moiseenko V Correa C Pierce LJ Allen AM Radiation dose-volume effects in the heart. *Int J Radiat Oncol Biol Phys.* (2010) 76:S77–85. 10.1016/j.ijrobp.2009.04.093 20171522

[35] Filippi AR Meregalli S Di Russo A Levis M Ciammella P Buglione M Fondazione Italiana Linfomi (FIL) expert consensus on the use of intensity-modulated and image-guided radiotherapy for Hodgkin’s lymphoma involving the mediastinum. *Radiat Oncol.* (2020) 15:62. 10.1186/s13014-020-01504-8 32164700 PMC7066773

[36] Haasbeek CJA Lagerwaard FJ Slotman BJ Senan S. Outcomes of stereotactic ablative radiotherapy for centrally located early-stage lung cancer. *J Thorac Oncol.* (2011) 6:2036–43. 10.1097/JTO.0b013e31822e71d8 21892102

[37] Graeff C Bert C. Noninvasive cardiac arrhythmia ablation with particle beams. *Med Phys.* (2018) 45:e1024–35. 10.1002/mp.12595 30421810

[38] Bert C Engenhart-Cabillic R Durante M. Particle therapy for noncancer diseases. *Med Phys.* (2012) 39:1716–27. 10.1118/1.3691903 22482597

[39] Moher D Liberati A Tetzlaff J Altman DG Altman D Antes G Preferred reporting items for systematic reviews and meta-analyses: the PRISMA statement. *PLoS Med.* (2009) 6:e1000097. 10.1371/journal.pmed.1000097 19621072 PMC2707599

[40] Page MJ Mckenzie JE Bossuyt PM Boutron I Hoffmann T Mulrow CD Updating the PRISMA reporting guideline for systematic reviews and meta-analyses: study protocol. Published online. (2018) 14:2018.

[41] Sharma A Wong D Weidlich G Fogarty T Jack A Sumanaweera T Noninvasive stereotactic radiosurgery (CyberHeart) for creation of ablation lesions in the atrium. *Heart Rhythm.* (2010) 7:802–10. 10.1016/j.hrthm.2010.02.010 20156591

[42] Gardner EA Sumanaweera TS Blanck O Iwamura AK Steel JP Dieterich S *In vivo* dose measurement using tlds and MOSFET dosimeters for cardiac radiosurgery. *J Appl Clin Med Phys.* (2012) 13:190–203. 10.1120/jacmp.v13i3.3745 22584173 PMC5716562

[43] Zei PC Wong D Gardner E Fogarty T Maguire P. Safety and efficacy of stereotactic radioablation targeting pulmonary vein tissues in an experimental model. *Heart Rhythm.* (2018) 15:1420–7. 10.1016/j.hrthm.2018.04.015 29678783

[44] Chang JH Cha MJ Seo JW Kim HJ Park SY Kim BH Feasibility study on stereotactic radiotherapy for total pulmonary vein isolation in a canine model. *Sci Rep.* (2021) 11:12369. 10.1038/s41598-021-91660-y 34117284 PMC8196028

[45] Blanck O Bode F Gebhard M Hunold P Brandt S Bruder R Dose-escalation study for cardiac radiosurgery in a porcine model. *Int J Radiat Oncol Biol Phys.* (2014) 89:590–8. 10.1016/j.ijrobp.2014.02.036 24751407

[46] Bode F Blanck O Gebhard M Hunold P Grossherr M Brandt S Pulmonary vein isolation by radiosurgery: implications for non-invasive treatment of atrial fibrillation. *Europace.* (2015) 17:1868–74. 10.1093/europace/euu406 25736725

[47] Maguire PJ Gardner E Jack AB Zei P Al-Ahmad A Fajardo L Cardiac radiosurgery (CyberHeartTM) for treatment of arrhythmia: physiologic and histopathologic correlation in the porcine model. *Cureus.* (2011) 3:e32. 10.7759/cureus.32

[48] Consorti R Petrucci A Fortunato F Soriani A Marzi S Iaccarino G *In vivo* dosimetry with MOSFETs: dosimetric characterization and first clinical results in intraoperative radiotherapy. *Int J Radiat Oncol Biol Phys.* (2005) 63:952–60. 10.1016/j.ijrobp.2005.02.049 16199324

[49] Scalchi P Righetto R Cavedon C Francescon P Colombo F. Direct tumor *in vivo* dosimetry in highly-conformal radiotherapy: a feasibility study of implantable MOSFETs for hypofractionated extracranial treatments using the Cyberknife system. *Med Phys.* (2010) 37:1413–23. 10.1118/1.3315370 20443463 PMC2826391

[50] Blanck O Ipsen S Chan MK Bauer R Kerl M Hunold P Treatment planning considerations for robotic guided cardiac radiosurgery for atrial fibrillation. *Cureus.* (2016) 8:e705. 10.7759/cureus.705 27588226 PMC4999353

[51] Gardner EA Weidlich GA. Analysis of dose distribution in the heart for radiosurgical ablation of atrial fibrillation. *Cureus.* (2016) 8:e703. 10.7759/cureus.703 27610282 PMC4999153

[52] Xia P Kotecha R Sharma N Andrews M Stephans KL Oberti C A treatment planning study of stereotactic body radiotherapy for atrial fibrillation. *Cureus.* (2016) 8:e678. 10.7759/cureus.678 27563504 PMC4985047

[53] Lydiard S Caillet V Ipsen S O’Brien R Blanck O Poulsen PR Investigating multi-leaf collimator tracking in stereotactic arrhythmic radioablation (STAR) treatments for atrial fibrillation. *Phys Med Biol.* (2018) 63:195008. 10.1088/1361-6560/aadf7c 30189419

[54] Park C Papiez L Zhang S Story M Timmerman RD. Universal survival curve and single fraction equivalent dose: useful tools in understanding potency of ablative radiotherapy. *Int J Radiat Oncol Biol Phys.* (2008) 70:847–52. 10.1016/J.IJROBP.2007.10.059 18262098

[55] Ipsen S Blanck O Oborn B Bode F Liney G Hunold P Radiotherapy beyond cancer: target localization in real-time MRI and treatment planning for cardiac radiosurgery. *Med Phys.* (2014) 41:120702. 10.1118/1.4901414 25471947

[56] Armanious MA Mohammadi H Khodor S Oliver DE Johnstone PA Fradley MG. Cardiovascular effects of radiation therapy. *Curr Probl Cancer.* (2018) 42:433–42. 10.1016/j.currproblcancer.2018.05.008 30006103

[57] Kottkamp H Piorkowski C Tanner H Kobza R Dorszewski A Schirdewahn P Topographic variability of the esophageal left atrial relation influencing ablation lines in patients with atrial fibrillation. *J Cardiovasc Electrophysiol.* (2005) 16:146–50. 10.1046/j.1540-8167.2005.40604.x 15720452

[58] Constantinescu A Lehmann HI Packer DL Bert C Durante M Graeff C. Treatment planning studies in patient data with scanned carbon ion beams for catheter-free ablation of atrial fibrillation. *J Cardiovasc Electrophysiol.* (2016) 27:335–44. 10.1111/jce.12888 26638826

[59] Richter D Lehmann HI Eichhorn A Constantinescu AM Kaderka R Prall M ECG-based 4D-dose reconstruction of cardiac arrhythmia ablation with carbon ion beams: application in a porcine model. *Phys Med Biol.* (2017) 62:6869–83. 10.1088/1361-6560/aa7b67 28644151

[60] Lehmann HI Graeff C Simoniello P Constantinescu A Takami M Lugenbiel P Feasibility study on cardiac arrhythmia ablation using high-energy heavy ion beams. *Sci Rep.* (2016) 6:38895. 10.1038/srep38895 27996023 PMC5171237

[61] Ren XY He PK Gao XS Zhao ZL Zhao B Bai Y Dosimetric feasibility of stereotactic ablative radiotherapy in pulmonary vein isolation for atrial fibrillation using intensity-modulated proton therapy. *J Appl Clin Med Phys.* (2021) 22:79–88. 10.1002/acm2.13239 33817981 PMC8130224

[62] Monroy E Azpiri J De La Peña C Cardona C Hinojosa M Zamarripa R Late gadolinium enhancement cardiac magnetic resonance imaging post robotic radiosurgical pulmonary vein isolation (RRPVI). First case in the world. *Cureus.* (2016) 8:e738. 10.7759/cureus.738 27660737 PMC5025292

[63] Qian PC Azpiri JR Assad J Gonzales Aceves EN Cardona Ibarra CE de la Pena C Noninvasive stereotactic radioablation for the treatment of atrial fibrillation: first-in-man experience. *J Arrhythmia.* (2020) 36:67–74. 10.1002/joa3.12283 32071622 PMC7011819

[64] Shoji M Inaba K Itami J Hamada M Okamoto H Iwasa T Advantages and challenges for noninvasive atrial fibrillation ablation. *J Interv Card Electrophysiol.* (2021) 62:319–27. 10.1007/s10840-020-00904-w/Published 33106957

[65] Rahimian J Torossian A Shenasa MA. Feasibility and proof of concept dosimetric study of noninvasive radiosurgical ablation of pulmonary vein antra to treat atrial fibrillation. *Int J Radiat Oncol.* (2016) 96:E635–6. 10.1016/j.ijrobp.2016.06.2220

[66] Gregucci F Di Monaco A Bonaparte I Surgo A Caliandro M Carbonara R Linac based stereotactic arrhytmia radioablation for atrial fibrillation: preliminary evaluation. In: *Proceedings of the AIRO – XXXI Conference* (2021).

[67] Kuck KH Hoffmann BA Ernst S Wegscheider K Treszl A Metzner A Impact of complete versus incomplete circumferential lines around the pulmonary veins during catheter ablation of paroxysmal atrial fibrillation: results from the gap-atrial fibrillation-German atrial fibrillation competence network 1 trial. *Circ Arrhythm Electrophysiol.* (2016) 9:e003337. 10.1161/CIRCEP.115.003337 26763226

[68] Nishida K Michael G Dobrev D Nattel S. Animal models for atrial fibrillation: clinical insights and scientific opportunities. *Europace.* (2010) 12:160–72. 10.1093/europace/eup328 19875395

[69] Fowler JF. The linear-quadratic formula and progress in fractionated radiotherapy. *Br J Radiol.* (1989) 62:679–94. 10.1259/0007-1285-62-740-679 2670032

[70] Grimm J LaCouture T Croce R Yeo I Zhu Y Xue J. Dose tolerance limits and dose volume histogram evaluation for stereotactic body radiotherapy. *J Appl Clin Med Phys.* (2011) 12:267–92. 10.1120/jacmp.v12i2.3368 21587185 PMC5718687

[71] Abelson JA Murphy JD Loo BW Chang DT Daly ME Wiegner EA Esophageal tolerance to high-dose stereotactic ablative radiotherapy. *Dis Esophagus.* (2012) 25:623–9. 10.1111/j.1442-2050.2011.01295.x 22168251

[72] Zei PC Soltys S. Ablative Radiotherapy as a noninvasive alternative to catheter ablation for cardiac arrhythmias. *Curr Cardiol Rep.* (2017) 19:79. 10.1007/s11886-017-0886-2 28752279 PMC5532420

[73] Ipsen S Blanck O Lowther NJ Liney GP Rai R Bode F Towards real-time MRI-guided 3D localization of deforming targets for non-invasive cardiac radiosurgery. *Phys Med Biol.* (2016) 61:7848–63. 10.1088/0031-9155/61/22/7848 27779127

[74] Lydiard S Pontré B Hindley N Lowe BS Sasso G Keall P. MRI-guided cardiac-induced target motion tracking for atrial fibrillation cardiac radioablation: MRIg tracking: AF CR targets. *Radiother Oncol.* (2021) 164:138–45. 10.1016/j.radonc.2021.09.025 34597739

[75] LaRiviere MJ Santos PMG Hill-Kayser CE Metz JM. Proton therapy. *Hematol Oncol Clin North Am.* (2019) 33:989–1009. 10.1016/j.hoc.2019.08.006 31668216

[76] Amino M Yoshioka K Kamada T Furusawa Y. The potential application of heavy ion beams in the treatment of arrhythmia: the role of radiation-induced modulation of connexin43 and the sympathetic nervous system. *Int J Part Ther.* (2019) 5:140–50. 10.14338/IJPT-18-00022.1 31773026 PMC6871596

[77] Baldi A Abbate A Bussani R Patti G Melfi R Angelini A Apoptosis and post-infarction left ventricular remodeling. *J Mol Cell Cardiol.* (2002) 34:165–74. 10.1006/jmcc.2001.1498 11851356

[78] Kajstura J Cheng W Reiss K Clark WA Sonnenblick EH Krajewski S Apoptotic and necrotic myocyte cell deaths are independent contributing variables of infarct size in rats. *Lab Invest.* (1996) 74:86–107. 8569201

[79] Haskova J Jedlickova K Cvek J Knybel L Neuwirth R Kautzner J. Oesophagopericardial fistula as a late complication of stereotactic radiotherapy for recurrent ventricular tachycardia. *Europace.* (2022). euab326. 10.1093/europace/euab326 35138366

[80] Kovacs B Mayinger M Schindler M Steffel J Andratschke N Saguner AM. Stereotactic radioablation of ventricular arrhythmias in patients with structural heart disease – a systematic review. *Radiother Oncol.* (2021) 162:132–9. 10.1016/j.radonc.2021.06.036 34233215

[81] Mustapha B Aydogan B Nolen J Nassiri A Noonan J. Prospects for an advanced heavy ion therapy center in the Chicago area. *Proceedings of the AIP Conference Proceedings 2160, 050009.* Melville, NY: API Publishing (2019).

