# Supplementary material for: Stereotactic Radiotherapy Ablation and Atrial Fibrillation: Technical Issues and Clinical Expectations Derived From a Systematic Review
Source: Front Cardiovasc Med. 2022 May 3;9:849201. doi: 10.3389/fcvm.2022.849201 (PMC9110686; doi:10.3389/fcvm.2022.849201)
Supplement: Supplementary file 2 [file Data_Sheet_2.pdf]

SUPPLEMENTARY MATERIAL 2

SUMMARY AND DEFINITION OF THE RADIATION ONCOLOGY-RELATED TERMS

**4DCT**	Four-Dimensional Computed Tomography	Method for acquiring CT images which accounts for patients' respiratory motion, especially useful for thoracic indications. The name refers to the inclusion of time (i.e. respiratory cycle) as a fundamental component of the acquisition process.
**α/β**	α/β Ratio	The ratio of "intrinsic radiosensitivity" to "repair capability" of a specified tissue (both espressed in Gray, Gy), either tumoral or healthy
**BED**	Biological Effective Dose	A measure of the true biological dose delivered by a particular combination of dose per fraction and total dose to a particular tissue characterized by a specific α/β ratio
**BP**	Bragg Peak	The point in space where a ionizing particle deposits its maximum energy during its travel through matter; it is specific for each particle (e.g. protons, carbon ions).
**CK**	Cyberknife	Linear accelerator with a robotic arm that permits stereotactic radiosurgery.
**CLA**	Conventional Linear Accelerator	A machine used to deliver external beam radiation treatments to the intended treatment target
**HT**	Helical Tomotherapy	A type of CLA whose main characteristic is that the radiation source around the patient in a spiral (or helical) pattern. It is used to deliver an intensity modulated treatment
**IMPT**	Intensity - Modulated Proton Therapy	A type of proton-based radiation therapy that permits to conform proton beam radiations to the shape of the target, and of healthy structures, in order to optimize both target coverage and sparing of non-target volumes
**IMRT**	Intensity - Modulated Radiation Therapy	A type of radiation therapy that permits to conform proton beam radiations to the shape of the target, and of healthy structures, in order to optimize both target coverage and sparing of non-target volumes
**MOSFET**	Metal Oxide Semiconductor Field Effect Transistor	A type of insulated-gate field-effect transistor that is fabricated by the controlled oxidation of a semiconductor, typically silicon
**MRI-Linac**	Magnetic Resonance Imaging-Linear Accelerator	A type of linear accelerator in which radiation delivery is guided through the real-time acquisition of magnetic resonance imaging
**OAR**	Organs at risk	Any healthy tissue/ organ located near the clinical target volume, whose irradiation could cause tissue/ organ damage, and eventually the onset of radiation-induced clinical side effects.
**PBT**	Particle Beam Therapy	Alternative treatment modality which uses beams of protons or other charged particles instead of photons
**RBE**	Relative Biological Effectiveness	The ratio of the absorbed dose of a reference radiation to the absorbed dose of the specific examined radiation, that is required to attain the same level of biological effect
**STAR**	STereotactic Arrhythmia Radioablation	A type of radiotherapy treatment used for ablating arrhythmia foci, based on a stereotactic technique (namely, the delivery of 1-5, high-dose fractions, in highly-conformed multiple beams)
**TV**	Target Volume	The target of any radiation treatment, and the reference volume for any dose prescription in radiation therapy

**VMAT**	Volumetric Modulated Arc Therapy	A type of IMRT consisting in the rotation of the radiation source around the patient, in an arc shape
**XRT**	Photon RadioTherapy	A type of radiation therapy that uses x-rays or gamma rays accelerated from a dedicated machine called linear accelerator
